# Round the Hospitals

**Published:** 1921-01-29

**Authors:** 


### ROUND THE HOSPITALS.

The Queen has graciously consented to lay the foundation-stone of the first block of buildings in the new Nurses' Home which is to bear her name at St. Bartholomew's Hospital on February 17. There is great satisfaction among Devonshire nurses that already one "Devonian room" in Queen Mary's Nurses' Home has been subscribed for in the county, while the subscription list for a second is already mounting up. Each room is estimated to cost £350, and already thirty-three have been named, some by localities subscribing for this purpose, and others as memorials to fallen officers and men. Donations should be sent to Lieut.-Col. W. McAdam Eccles, St. Bartholomew's Hospital, E.C. 1.

The annual prize-giving at the Leeds General Infirmary will long be remembered. The prizes were presented by Mrs. Charles Lupton, wife of the hon. treasurer of the hospital. Mr. Charles Lupton gave an interesting review of the training-school during the past seventy-six years. In the eighteenth century no nurse was accepted under the age of forty-three, the matron received £10 a year and the nurses £5, but even then the institution had a European reputation. Mr. Lupton concluded with the impressive announcement that they had asked the University of Leeds to establish a diploma in nursing. He was very sanguine that the University would adopt the proposal, in which case they would be the first university in the country to do so. The address given to the assembled nurses by Sir Berkeley Moynihan, K.C.M.G., C.B., a member of the Nurses' Training-School Committee at the infirmary, was of that kind which is for all time. None who were privileged to be present on the occasion can ever forget the moving words in which he painted the difficulties, the trials, and the triumphs of the nurse. We reprint on page 419 the entire address, and could wish that it could be made accessible in some attractive form for distribution to probationers and nurses in other training-schools. It is not surprising to find that Sir Berkeley Moynihan is a great supporter of the effort now in progress to raise the status of the nursing profession. He believes in the grading of nurses by qualifications, as there is a grading of medical men, and that the sister in charge of a ward, the theatre sister, or the matron in a teaching hospital should bear evidence in their qualification of longer study and more careful training, as it is in the case of the medical staff in a teaching hospital.

---

The adverse report of Miss Wamsley, inspector, of the Ministry of Health, on the nursing at the Islington Infirmary has created a great sensation in the institution, and, indeed, in the borough. Miss Wamsley found things very unsatisfactory in some of the wards from the nursing point of view, and performed her duty in reporting this to the Ministry of Health. Such inspections would be a mere waste of public money if the inspectors did not nerve themselves to speak the truth. Miss Wamsley formed the opinion that the nurses in these particular wards (it should be observed that she did not complain of the institution generally) appeared to be uninterested in their work—tired and jaded, in fact—and traced the cause to "late hours and constant excitement." She asserted, moreover, that some of the staff appeared to have no regard for the inconvenience and extra work caused when they fail to return to work at the appointed time. A deputation from the staff has waited on the Board of Guardians, denying the allegations and indignantly protesting. The chairman has issued a formal denial on behalf of the Board, but we are glad to note that a thorough investigation is to be made into the circumstances referred to by Miss Wamsley. The members of the staff not implicated in this censure ought to be definitely cleared.

The shortage of candidates for hospitals continues to be acutely felt at Bristol, and came under notice lately, when a letter from the Ministry of Health relating to the relief of unemployment came before the Health Committee. The chairman (Mr. H. J. Maggs) asked that publicity should be given to the fact that they could find work for a number of young women as nurses in their various institutions. The work of the hospitals was crippled because the staffs were inadequate. They could not open some of their wards for the reception of urgent cases on account of the dearth of nurses. It was further stated that they could at once double both the nursing and domestic staffs in Bristol institutions if they could get women to come forward. There would seem to be need for a well-planned publicity campaign in Bristol and the outlying districts. Something more than merely accentuating the fact of the scarcity is required, for persons looking for a career instinctively avoid an opening they are told is unpopular unless its real advantages are brought home to them.

---

The South Wales Nursing Association has been in existence for ten years, has opened up nearly a hundred nursing societies within its sphere, and given free training to over a hundred district midwives. It has now ceased to exist and has handed over its duties to the county nursing associations newly formed in Pembroke, Brecon, Radnor, and Glamorganshire, each with its own county superintendent. Carmarthen and Cardiganshire will soon also have their own associations. Miss Crowther, who has been superintendent of the South Wales Nursing Association from the beginning and the mainspring of its successful operations, is retiring from the nursing profession to be married. It has been a good piece of pioneer work accomplished.

---

A meeting of the General Nursing Council will be held on Wednesday, February 2, at 10.30 a.m. at the Ministry of Health, Whitehall.

---

The poor of Swansea, it appears, are without nurses at present—a state of things which surely will not long be allowed to exist in a wealthy town, the centre of the Welsh metal industries. Up to recently the nursing of the poor has been undertaken by the Queen's Nursing Association, which, however, has felt compelled to relinquish the work owing to lack of financial support. The plight of the sick poor was brought up at a meeting of the Guardians' Finance Committee on Thursday, when one of the lady members pleaded the cause of the unnursed very powerfully, and proposed that a temporary nurse be engaged. A counter-suggestion, that the Queen's Association be asked what additional subscriptions they needed in order to induce them to continue their local work, seemed to find more favour, however, and eventually it was decided to ask the Association whether they would be prepared to "carry on" for an increased contribution from the Guardians of £50, making £200 per annum in all. The next word lies with the Association, therefore.

